# Adoption, Acceptability, and Accuracy of an Online Clinical Trial Matching Website for Breast Cancer

**DOI:** 10.2196/jmir.1855

**Published:** 2012-07-11

**Authors:** Ellyn Cohen, Jeff Belkora, Joanne Tyler, Joan Schreiner, Mary Jo Deering, Lakshmi Grama, Brenda Duggan, Julie Illi, Julia Pederson, Aprajita Anand, Alexandra Teng, Erin McCreary, Dan Moore, Debu Tripathy, Michael Hogarth, Morton Lieberman, John Park, Laura Esserman

**Affiliations:** ^1^Carol Franc Buck Breast Care CenterUniversity of California San FranciscoSan Francisco, CAUnited States; ^2^Patient AdvocateSan Francisco, CAUnited States; ^3^Center for Biomedical Informatics and Information TechnologyNational Cancer InstituteRockville, MDUnited States; ^4^Office of Communications and EducationNational Cancer InstituteRockville, MDUnited States; ^5^California Pacific Medical Center Research InstituteSan Francisco, CAUnited States; ^6^Department of Pathology and Laboratory MedicineUniversity of California DavisDavis, CAUnited States; ^7^Department of PsychiatryUniversity of California San FranciscoSan Francisco, CAUnited States

**Keywords:** Breast cancer, clinical trials, cancer information

## Abstract

**Background:**

Less than 5% of breast cancer patients participate in clinical trials. To increase patients’ awareness and access to trials, we created BreastCancerTrials.org, a clinical trial matching website. BreastCancerTrials.org matched patients to trials based on their self-reported breast cancer history. It also provided a messaging platform through which patients could self-refer themselves to participating research sites.

**Objective:**

To assess adoption by research sites, acceptability to patients, and patients’ accuracy in providing information to BreastCancerTrials.org.

**Methods:**

We approached 13 research sites in Northern California to list their trials on BreastCancerTrials.org. For adoption, we examined the willingness of contacted research sites to collaborate with BreastCancerTrials.org. For acceptability, we analyzed usage statistics of visitors who completed the BreastCancerTrials.org health history questionnaire in the first 14 months after launch and surveyed users who visited the website during its first year about their experience. For accuracy, we compared the self-reported health history of 20 patients against their medical records. The health history questionnaire was divided into four sections: About Me, personal information including date of birth and sex; My Health as of Today, current status including cancer stage, menopausal status, and sites with evidence of disease; My Cancer, diagnostic information such as hormone and human epidermal growth factor receptor 2 status; and My Treatment, an itemized record of past treatment including responses to therapy.

**Results:**

A total of 12 sites contributed 55 trials. Regarding acceptability, 733 visitors registered on the website; 428 reported their health history; and 407 matched to at least one trial. Of 375 patients who were sent a survey, 75 responded (20%); 23 of the 75 (31%) contacted a research site, 12 of the 23 (52%) were eligible for a trial, and 5 of the 12 (42%) reported enrolling. As for accuracy, 20 clinic visitors reported 1456 health history items, 1324 of which matched their clinic record (90.93%).

**Conclusions:**

BreastCancerTrials.org was adopted by research sites. Patients found it acceptable and were able to provide accurate information for trial matching. Based on our findings, we launched an upgraded version of BreastCancerTrials.org as a national service in October 2008.

## Introduction

Most treatments used for breast cancer today are based on the results of clinical trials. Among the first pivotal trials that changed practice was NSABP B-06 [1], which demonstrated that lumpectomy with lymph node removal and radiation, compared with mastectomy, is as effective at reducing distant recurrence and prolonging survival. Clinical trials have enabled the field to progress by providing better options and more clarity to patients on the risks and benefits of cytotoxic chemotherapy, targeted agents, and local–regional strategies. However, less than 5% of women with breast cancer participate in clinical trials, limiting the pace at which researchers can test new and alternative treatment and prevention strategies [2].

Joining a clinical trial requires awareness of opportunities, alignment of opportunities with the individual’s goals for treatment, and access to research sites that are conducting trials. Patients’ lack of awareness about trials is a major barrier to accrual and the timely completion of clinical trials [3,4]. Most patients learn about trials from their physicians and are more apt to participate if physicians support their patients’ involvement. However, many physicians are unaware of opportunities, do not have the time to review inclusion requirements, or make erroneous assumptions about their patients’ potential eligibility [5,6].

Various initiatives are under way to address barriers to clinical trial participation. Several organizations are conducting awareness campaigns for the general public, as well as underserved populations not typically represented in clinical trials. In the United States, these include the Coalition of Cancer Cooperative Groups, C-Change, the Lance Armstrong Foundation, the Education Network to Advance Cancer Clinical Trials, the Center for Information and Study on Clinical Research Participation, and Eliminating Disparities in Clinical Trials. Additionally, several groups have developed prototype information systems for matching patients’ histories to trial eligibility criteria at the point of care [7-11].

Simultaneously, patients are turning to the Internet for health care-related information [12]. Several resources are available to assist such individuals in finding a clinical trial as described in a 2008 review by the US National Cancer Institute [13]. Patients can search for, evaluate, and locate clinical trials on government-sponsored websites (www.clinicaltrials.gov or www.cancer.gov); on a site sponsored by the nonprofit Coalition of Cancer Cooperative Groups (www.cancertrialshelp.org); and on for-profit websites (eg, www.emergingmed.com) [14-17].

One potential problem with these websites is that they rely on research site locations and contact information listed in government databases. As a consequence, one study pointed out that these sources sometimes feature “out-of-date” research site information, with “incorrect contact information and trial listings” [18].

In 1999, two women with breast cancer, Joan Schreiner and Joanne Tyler, conceived of an independent, nongovernmental, nonprofit clinical trial matching service, available over the Internet, which would enable breast cancer patients to find trials personalized to their situation. With their input, clinical investigators from the Center of Excellence for Breast Cancer Care at the University of California, San Francisco (UCSF) and health and information specialists from the National Cancer Institute developed the concept into a website named BreastCancerTrials.org. By design, BreastCancerTrials.org worked with research sites (ie, the hospitals and clinics that enroll patients) to maintain an up-to-date list of open trials. BreastCancerTrials.org also facilitated a process by which individuals communicate directly with research sites about trials of interest. This design addressed the need to align opportunities with individual conditions and goals, and for patients to access a research site that is implementing a desired trial protocol.

BreastCancerTrials.org launched in 2005 as a regional research study featuring trials in the San Francisco Bay Area. We were generally interested in whether research sites would adopt or collaborate with BreastCancerTrials.org, whether patients would accept the website requirements for matching, and whether patients could accurately report detailed personal health information.

## Methods

### Study Questions

We formulated 3 specific study questions. First, we asked whether research sites would adopt and collaborate with a clinical trial matching service that requires them to update information about their trials and accept messages from patients. Second, we asked whether patients would complete a detailed online health history, match to a trial, contact a trial site, enroll in a trial, and be satisfied with their experience. Third, we asked whether patients would enter correct information into the BreastCancerTrials.org health history questionnaire.

### Study Design

We answered the study questions through a descriptive case study. We launched BreastCancerTrials.org as the intervention, and monitored the behaviors of research sites and patients through data collection mechanisms described below. We sought and obtained approval from the UCSF Committee on Human Research and administered an approved outreach, recruitment, and consent process for both research site investigators and patients who participated in the study.

The 3 study questions involved different dimensions of BreastCancerTrials.org. We therefore present our methods, results, and conclusions in a parallel structure. Specifically, for each section of the ensuing report, we have separate paragraphs for the adoption, acceptability, and accuracy questions.

### Intervention

**Figure 1:**
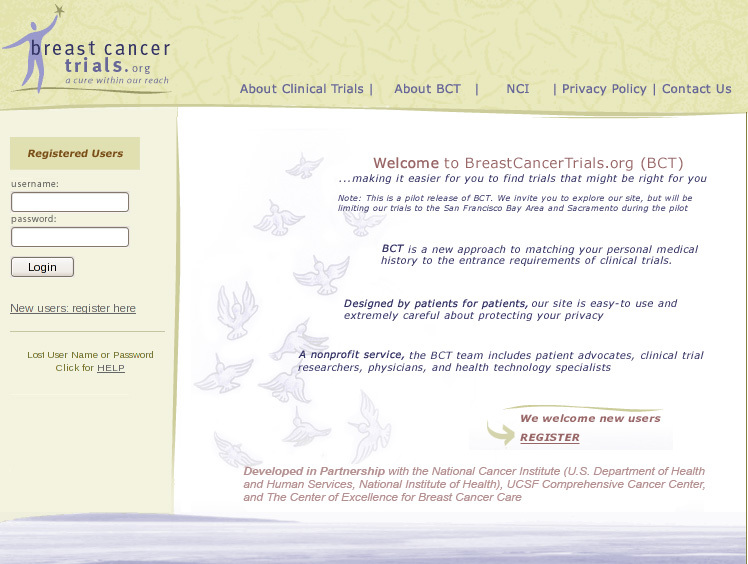
Homepage of BreastCancerTrials.org.

Overall, the intervention consisted of research sites’ and patients’ use of BreastCancerTrials.org. (Figure 1).

Research sites and patients interacted with different components of the website, so the intervention varied as described below.

For the question of research site adoption, to appear in BreastCancerTrials.org, research sites had to submit their protocols and contact information and review our coding of the eligibility criteria into a machine-readable format. We solicited study protocols prior to the launch of BreastCancerTrials.org and on a monthly basis afterward. We coded protocols and alerted research sites when their trials were uploaded to the website. At this point site personnel were invited to review coding and provide feedback.

Regarding acceptability to patients, patients using BreastCancerTrials.org were matched to relevant trials based on the completion of a self-reported health history questionnaire. The questionnaire functioned as an intervention in our study, since it was a component of a health service for clinical trial matching.

**Figure 2:**
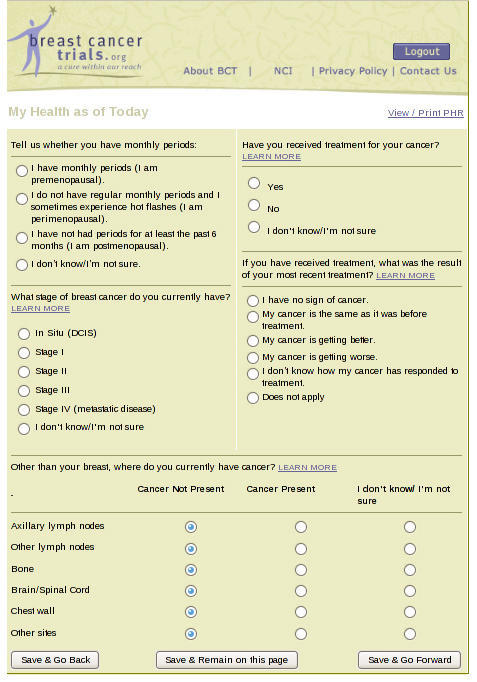
A section from the My Health portion of the BreastCancerTrials.org health history questionnaire.

The health history questionnaire was divided into four sections, three of which captured a fixed number of items: About Me, 12 items, including patients’ date of birth and sex; My Health as of Today, 15 items, including current cancer stage, menopausal status, and sites with evidence of disease (Figure 2); and My Cancer, 13 items, including estrogen, progesterone, and human epidermal growth factor receptor 2 (HER2/neu) status. In the fourth section, My Treatment, patients entered their past surgeries as well as radiation, chemo-, hormonal, targeted/biological, and bisphosphonate therapies. After selecting a treatment, patients also provided additional information, for example, whether the treatment was received in the neoadjuvant, adjuvant, or metastatic setting and their response to each therapy. The number of items for My Treatment had no upper limit because respondents could add as many treatments as they had experienced including, for example, multiple cycles of chemotherapy. The matching engine, caMatch, was developed in collaboration with the National Cancer Institute, based on a prototype built at UCSF. It compared patients’ health history questionnaires with trial eligibility requirements in the coded protocols. The matching engine provided a report of relevant trials along with the contact information for the enrollment coordinator at each trial site. An online messaging platform was included as part of the matching service. It allowed patients to contact a research site about their interest in a specific matched trial and to invite a coordinator to view their online health history. Patients’ consent was required for BreastCancerTrials.org to make the history available to a specific coordinator for online viewing; patients’ health summaries were never sent to coordinators via email.

In addressing the accuracy question, the intervention consisted of asking UCSF breast cancer patients to register on the website and complete the health history questionnaire. Subsequently we evaluated the accuracy of each patient-reported health history by comparing it with the patient’s official medical record, as described in the outcomes section below.

### Sample

For the question about adoption by research sites, from February 2005 to May 2005, author EC identified a convenience sample of 13 institutions in the 9-county San Francisco Bay Area and Sacramento, where she had found investigators who were conducting breast cancer clinical trials. The research sites included academic, private practice, and managed care settings. EC invited these research sites to submit descriptions of their open trials, including eligibility criteria, prior to the regional launch of BreastCancerTrials.org and on a monthly basis thereafter.

For the question about acceptability to patients, we included all people who registered on the BreastCancerTrials.org website in the first 14 months after launch (between June 2005 and September 2006) in our analysis of website usage. Between July 1, 2005 and June 30, 2006, we sent emails inviting website registrants who had completed the health history questionnaire in the first 12 months after launch to respond to our survey asking for user ratings of the website.

For the question about patients’ accuracy, between April 25, 2006 and March 29, 2007, we invited a convenience sample of patients who had completed at least one episode of treatment at the UCSF Carol Franc Buck Breast Care Center. Through a process approved by our institutional review board, a research assistant periodically reminded the 12 attending physicians at the Breast Care Center of the opportunity to refer patients to this study. The physicians served as referral sources but were not otherwise involved in the study. The research assistant went to the clinic whenever patients referred by their physicians were being seen. The research assistant approached such patients until we had accrued our target of 20 who had completed the study requirements by filling out the health history questionnaire online.

### Measures and Data Collection Procedures

We measured research site adoption by counting the number of sites that contributed trials to the regional launch of BreastCancerTrials.org, and by tallying the number of trials each research site submitted and kept up-to-date in the BreastCancerTrials.org repository of trials.

We measured acceptability to patients of BreastCancerTrials.org by observing the number of (1) patients registering, (2) consenting to the study and starting the health history questionnaire, and (3) completing the health history questionnaire. We measured patients’ reactions to BreastCancerTrials.org by administering a follow-up, anonymous survey with 13 items, 6 of which were relevant to our study questions. We therefore analyzed responses to the following 6 summative evaluation items. A total of 3 yes/no items probed whether the patient contacted a research site after matching to a trial on the website; whether they were eligible for the trial on further review by the research site; and whether they ultimately enrolled in the trial. For these binary items, we counted the number and proportion of respondents answering affirmatively. A further 3 items addressed overall satisfaction; likelihood to recommend to friends or colleagues; and ease of navigation [19]. These were rated on a 10-point scale where 1 anchored the most negative response and 10 the most positive. We calculated the mean responses to each of these items individually. The remaining 7 survey questions were not directly related to our study questions and we did not analyze them for this study.

We measured accuracy of patient-reported health history by comparing patients’ responses to the BreastCancerTrials.org health history questionnaire with the health history reflected in the patients’ medical charts. Patients filled out the health history questionnaire via the BreastCancerTrials.org website, using their own personal records and recollections. Meanwhile, a study coordinator (author AA or JP) abstracted data from each respondent’s medical chart and created a second, chart-based instance of the patient’s health history questionnaire. We treated this chart-based version of the health history as the reference standard (sometimes known as the gold standard). We calculated accuracy by forming a ratio. Where patients had either answered a question incorrectly or omitted an item that was reported in the chart, we defined the denominator as the total number of items reported in the chart-based health history, and defined the numerator as the number of responses where the patient and chart agreed. Where patients provided items that were not in the chart, we added the number of such items to the denominator, and kept the numerator as the number of responses where the patient and chart agreed. We omitted from the accuracy analysis 12 items in the About Me section of the survey, as we considered the patient’s current responses to these demographics questions definitive regardless of what the chart indicated. We also omitted 2 items from the My Cancer section, neither of which was used for matching. These were “Are tissue samples from your breast cancer available for further testing?” and the optional question “If you were positive for HER2/neu, what method was used to test it?”

### Analysis

We analyzed quantitative measures using descriptive statistics including counts, means, medians, and standard deviations. We analyzed qualitative measures, such as open-ended survey responses, by reading for themes and then discussing them among study authors until we arrived at a consensus interpretation.

## Results

Regarding research site adoption, we approached 13 San Francisco and Sacramento-area health care organizations. At each research site, we invited a clinical trials manager or physician in charge to coordinate submission of active breast cancer study protocols and institutional review board approval documents to our research team. Of the 13 research sites, 12 completed the requirements for participation, by providing sufficient information for our research team to code their trials using our structured data entry forms. Of the 12 research sites, 2 were academic medical centers, 6 were community hospitals, 1 was a participant in the National Cancer Institute-funded Community Clinical Oncology Program, 1 was a health maintenance organization, and 2 were private oncology practices (Table 1). These 12 research sites contributed all of their breast cancer protocols, representing 55 studies during the study period. The BreastCancerTrials.org team coded the trials in the BreastCancerTrials.org database and sent them to the research sites for review and approval. The research sites reported no disagreements with the BreastCancerTrials.org codes, so the team activated the trials on the production website. A total of 11 research sites remained in the study through its completion; 1 withdrew after the first year because the clinical trial manager submitting active study documents felt this required too much time.

With regard to patients using the site and matching to clinical trials, the population for this study question consisted of 733 patients registering on BreastCancerTrials.org between June 2005 and September 2006. Registration involved providing a name and email address, at which point patients could navigate the website and consent (or not) to use the matching service. Of 733 registrants, 614 (83.8%) consented to use the matching service, of whom 491 (80.0%) also reported demographics (Table 2).

We tracked website usage as an indication of BreastCancerTrials.org’s acceptability to patients. Of the 614 patients who registered and consented, 428 (69.7%) completed the minimum health history elements required to match to a trial. Of these 428, 407 (95.1%) matched to at least one trial. Of the 407 matched visitors, 70 (17%) connected with a research site through BreastCancerTrials.org’s Message Center.

Between July 1, 2005 and June 30, 2006, we sent email surveys to 375 website users who had completed the health history questionnaire during the first 12 months after launch, and invited them to respond anonymously. We found that 75 of 375 (20%) responded, and 23 of the respondents (31% ) reported contacting a research site. Among the 23 who contacted a research site, 12 (52%) reported being told they were eligible for a trial, and 5 of these 12 (42%) reported enrolling in a trial.

**Table 1:** Research sites providing trials at the launch of BreastCancerTrials.org.

Practice setting	Research site
Academic	University of California, San Francisco
	University of California, Davis
Community hospital	Sutter East Affiliated Hospitals (Sacramento)
	Sutter West Affiliated Hospitals (Alta Bates Summit Medical Center, California Pacific Medical Center, Mills-Peninsula Health Services)
	John Muir Health
Community Clinical Oncology Program	Bay Area Tumor Institute
Health maintenance organization	Northern California Kaiser Permanente
Private practice	Camino Alto (Peninsula)
	California Cancer Care (Marin)

**Table 2:** Demographics characteristics of respondents to acceptability and accuracy study questions.

Demographic characteristic		Acceptability analysis				Accuracy analysis	
		Website visitors reporting demographics (n = 491)		Online satisfaction survey respondents (n = 75)		Clinic-based health history questionnaire respondents (n = 20)	
		n	%	n	%	n	%
**Age range (years)**							
	<45	132	26.9%	15	20%	5	25%
	45–54	178	36.3%	23	31%	8	40%
	55–64	140	28.5%	17	23%	6	30%
	65+	39	8%	3	4%	1	5%
	No response	2	<1%	17	23%		
**Education**							
	Graduate/professional school	200	40.7%	31	41%	14	70%
	College graduate	163	33.2%	16	21%	6	30%
	<4 years of college	128	26.1%	7	9%	0	0%
	No response			21	28%		
**Race/ethnicity**							
	White	413	84.1%	49	65%	19	95%
	Hispanic/Latino	34	7%	2	3%	0	0%
	Black/African American	16	3%	2	3%	0	0%
	Asian/Pacific Islander	14	3%	2	3%	1	5%
	Other	14	3%	3	4%	0	0%
	No response			17	23%		

We present demographics reported by 75 survey respondents in Table 2 (middle columns). Respondents rated satisfaction with BreastCancerTrials.org at a mean level of 7 out of a maximum of 10. Respondents rated willingness to recommend BreastCancerTrials.org at a mean level of 7 out of 10. Finally, respondents rated the ease with which they completed the health history questionnaire at a mean level of 8 out of 10.

With regard to study questions about patients’ accuracy, 26 patients consented out of 57 approached (46%), 20 of whom completed the study requirements, for a response rate of 20 out of 57 (35%) among all approached, or a completion rate of 20 out of 26 (77%) among consenting patients. We invited them to fill out the health history questionnaire so we could compare their responses with the information in their medical record. Table 2 summarizes the demographic profiles of the final sample (last columns). These patients provided a total of 1456 items, matching the chart for 1324. Therefore, the overall accuracy rate was 90.93%. For the standard items in the My Health and the My Cancer sections, the accuracy rate was 469 out of 520 (90.2%). For the variable items in the My Treatment section, the accuracy rate was 855 out of 936 (91.4%).

 On an item-by-item level, accuracy ranged from 65% to 100% (see Table 3). On the low end of the range, 13 of the 20 (65%) respondents to the standard items correctly reported their progesterone status. At the high end of the range, respondents were 100% accurate in reporting their status with respect to pregnancy, current well-being, hypertension, cardiac arrhythmia, disease in other sites, local recurrence, type of bisphosphonate therapy taken, and type of biologic therapy taken.

**Table 3:** Accuracy of patients’ responses to health history questionnaire items, compared with a study coordinator’s abstraction of the data from the patients’ medical charts.

Item by website section		Total responses per Item	Items for which patient’s response matches chart	Accuracy rate
**My Health as of Today**				
	Menopausal status	20	19	95%
	Stage of current cancer	20	15	75%
	Treatment received? (yes/no)	20	20	100%
	Response to last treatment	20	15	75%
	Pregnant or nursing	20	20	100%
	Current well-being	20	20	100%
	Hypertension	20	20	100%
	Cardiac arrhythmia	20	20	100%
	Other medical problems	20	17	85%
	Disease in axillary lymph nodes	20	16	80%
	Disease in nonaxillary lymph nodes	20	19	95%
	Disease in bone	20	19	95%
	Disease in brain or spinal cord	20	19	95%
	Disease in chest wall	20	17	85%
	Disease in other sites	20	20	100%
	Subtotals	300	276	92.0%
**My Cancer**				
	Date of breast cancer diagnosis	20	19	95%
	Stage at diagnosis	20	18	90%
	Type of cancer	20	17	85%
	Estrogen receptor status	20	18	90%
	Progesterone receptor status	20	13	65%
	Positive lymph nodes	20	17	85%
	Sentinel node biopsy	20	17	85%
	Local recurrence	20	20	100%
	Metastasis	20	19	95%
	Inflammatory breast cancer	20	19	95%
	HER2/neu^a ^status	20	16	80%
	Subtotals	220	193	87.7%
**My Treatment**				
	Treatment modality (eg, surgery, radiation, chemotherapy)	96	84	88%
	Type of surgery (eg, mastectomy)	150	137	91.3%
	Location of radiation therapy (eg, breast, bone)	63	57	90%
	Type of hormone therapy (eg, tamoxifen)	91	90	99%
	Type of bisphosphonate therapy (eg, Zometa^b^)	12	12	100%
	Type of biologic therapy (eg, Herceptin^c^)	8	8	100%
	Type of chemotherapy (eg, paclitaxel)	340	328	96.5%
	Setting (neoadjuvant, adjuvant, metastatic)	88	81	92%
	Overall response (eg, no cancer, cancer progressed)	88	58	66%
	Subtotals	936	855	91.4%
Total		1456	1324	90.93%

^a ^Human epidermal growth factor receptor 2.

^b ^Generic name zoledronic acid.

^c ^Generic name trastuzumab.

While patients were accurate overall, we call attention to items for which patients had difficulty matching the chart. A total of 5 patients did not match the chart with respect to the stage of current cancer; 5 patients were not accurate in responding to response to last treatment; and 5 patients were inaccurate in responding to items about disease in lymph nodes, 4 of whom were wrong about axillary and 1 about nonaxillary lymph nodes.

## Discussion

### Interpretation and Analysis

Regarding the adoption by research sites, in the 3 months prior to launch, we attracted 12 of 13 research sites, 11 of which remained active in the study 1 year later. We had some concerns about whether the research sites would see collaborating with BreastCancerTrials.org as an added burden. Our experience suggests that research sites were willing to add BreastCancerTrials.org as an additional channel through which to reach potential study participants. We believe that in an environment of relatively low study participation, the value of adding participants is so high that research sites are motivated to spend additional time and other resources on exposing potential study candidates to the opportunity.

Regarding the acceptability to patients, almost all visitors (407/428, 95.1%) who provided sufficient information for matching did in fact match to at least one trial. Website visitors were satisfied, and 70 contacted a research site. These data support our hypothesis that as more people seek health information online [12], many could be attracted to websites dealing with clinical trials.

The demographics of participants who used the BreastCancerTrials.org matching tool were highly educated and predominantly white, not surprising given the demographics of people using the Internet to find health information in 2005 [12]. As more diverse populations use the Internet to access health information, further studies should explore whether they too are open to using BreastCancerTrials.org or similar clinical trial matching websites.

As for the accuracy of patients’ responses, the BreastCancerTrials.org health history questionnaire used in this study was notable for its level of detail. We presented a minimum of 40 items before patients even began filling out My Treatment, which could also require dozens of items depending on the complexity of treatment history. Our assumption that patients would be capable of answering detailed questions about their cancer treatment was validated by the high degree of accuracy when their answers were compared with their medical records. For example, 1 patient with metastases entered 10 chemotherapy regimens correctly.

We included both estrogen and progesterone receptor status in the health history questionnaire. However, during the study period, physicians in our clinic sometimes discussed estrogen or hormone receptor status without specifying the progesterone receptor status. We believe this may explain the discrepancy between patients’ ability to self-report their estrogen and progesterone receptor status. Given the emergence of triple-negative breast cancer as a distinct clinical entity, designers of clinical trial matching systems should include progesterone receptor status in their matching criteria and prompt patients to ask their physicians about it if necessary.

Of 20 patients, 5 (25%) were not accurate about their tumor response to prior treatment. In the adjuvant setting where there is no longer evidence of disease, it is easy to understand patients’ confusion with this question, as the possible answers focused on how the “cancer” responded to treatment. As we disseminated the website nationally, we recast the question. On all forms, we ask whether therapy was completed and, if not, why it was stopped. The choices for why it was stopped were “Stopped treatment due to side effects,” “Tumor occurred, recurred, or did not shrink with therapy,” and “I don’t’ know.” We standardized the question to fit across patient types and therapies.

We learned that, while 18 of 20 respondents matched their chart regarding stage at diagnosis, only 15 matched the chart when responding to “What stage of breast cancer do you have as of today?” One patient said in an unsolicited phone call to the BreastCancerTrials.org offices, “I’ve completed treatment. I don’t have cancer now.” We have since deleted this question from the BreastCancerTrials.org online health history as being confusing for people whose breast cancer tumor has been removed by local therapy.

### Connections to the Literature

We now turn to a discussion of how our findings complement or conflict with previous reports in the published literature.

With regard to our first study question, on adoption by research sites, we have not found published evidence regarding whether multiple research sites will list their trials in a common clinical trial matching website. Our positive finding extends the results of a study of clinical trial matching at a single location [18]. In that study, the University of Pennsylvania collaborated with EmergingMed to provide clinical trial matching for trials open at the University of Pennsylvania’s cancer center.

Regarding acceptability to patients, we did not know whether privacy issues or complexity would inhibit patients from providing the required health history data to BreastCancerTrials.org. A previously published usability study of BreastCancerTrials.org suggested that both could be barriers and tested design changes that addressed user concerns [20,21]. Our finding that 428 patients over 14 months reported their health history is consistent with the University of Pennsylvania’s experience, where they found that 627 patients provided sufficient information to match to clinical trials over a 15-month period [18]. Whereas the University of Pennsylvania system featured trials from one location across many cancer types, our system featured breast cancer trials only, but from many research site locations.

Regarding patient accuracy, we found 91% overall accuracy about details of the breast cancer diagnosis in a sample of patients undergoing breast cancer treatment at our medical center. No studies have reported patients’ accuracy for the other clinical trial matching systems. Other studies have found high overall accuracy of patients’ self-reports regarding the existence of a prior breast cancer and its treatment [22]. However, some patient subgroups are less accurate [22]. Also, patients may be less accurate in recollecting some aspects of their breast cancer history such as the stage of their cancer [23].

The American Society of Clinical Oncology has recommended that all patients be provided with a treatment summary as part of standard care for survivors [24]. This kind of summary would provide women with the data required for accurate clinical trial matching. In the future, the ability to capture these data in an electronic fashion would potentially enable an automated way for patients and providers to identify appropriate trials as a routine of practice.

### Limitations

This evaluation did not address some of the known threats to internal and external validity. For example, the proportion of research sites willing to provide their trial data may be different from a nationally representative sample.

In studying acceptability to patients, we invited only individuals who completed our health history to take the survey. With a response rate of 20%, we don’t know whether the majority of nonrespondents felt differently about their experience with BreastCancerTrials.org. In particular, although we learned that 5 respondents enrolled in a clinical trial, the overall enrollment rate among BreastCancerTrials.org users may have been higher or lower depending on whether respondents differed from nonrespondents. Sensitivity analysis suggests the enrollment rate may have varied from 1% (5/375 surveyed) to 7% (5/75 responding). In addition, our survey solicited only anonymous responses, so we could not follow up on responses. Thus, we learned that 12 of 23 people who contacted a research site were eligible for a study, but could not ascertain why the remaining 11 were ineligible.

We conducted the accuracy evaluation with a small sample of patients undergoing treatment at our academic medical center, not with website registrants. Our accuracy results may not reflect the accuracy of actual visitors to the website. We observed a 46% consent rate and a 77% completion rate for an overall 35% response rate relative to the patients we approached. This means that the majority of patients we approached declined to complete the health history questionnaire, limiting our ability to generalize our accuracy results. The accuracy results could also be misleading in that we treated the chart as being definitively correct. In fact, charts may be incomplete or erroneous.

For all of the study questions, our samples were highly educated relative to the general population. The clinic sample in the accuracy study was even better educated than the website registrants overall or than website registrants responding to our follow-up acceptability survey. This may limit the generalizability of our findings, as studies have shown that low-income and elderly populations face challenges to using electronic health tools [25].

### Conclusions

We conclude that BreastCancerTrials.org is a promising vehicle for connecting patients with opportunities to participate in clinical trials. Research sites were willing to register their trials, and over half of the patients who found BreastCancerTrials.org registered and completed a health history questionnaire. Patients also contacted research sites and some joined trials. The degree of accuracy in patients’ responses was high, which may be due to the fact that all of the patients had completed a 4-year college degree and 14 out of 20 (70%) reported postgraduate training. Still, based on our analysis of response patterns, we have been able to improve the way in which health history questions are asked. Researchers should continue to explore the question of accuracy with more diverse populations. People with lower health literacy may require assistance in providing accurate information to BreastCancerTrials.org or similar clinical trial matching tools.

Our study contributes new knowledge to the literature, including evidence that research sites will provide details of their open trials to third-party clinical trial matching systems; and patients can and will accurately fill out a detailed health history questionnaire as a means to matching to clinical trials online. Overall, this evaluation suggests that BreastCancerTrials.org could contribute to the public health agenda of making people more aware of clinical trials with a view to ultimately increasing enrollment.

As a result of this study, we secured funding to disseminate BreastCancerTrials.org so that it includes research sites across the United States. This is a reportable outcome in terms of implementation and maintenance of a research-initiated innovation. The nationwide service operates at www.BreastCancerTrials.org as a program of Quantum Leap Healthcare Collaborative, a nonprofit corporation [26]. Quantum Leap has successfully solicited philanthropic donations to maintain the clinical trial matching service and is exploring additional sources of recurring revenue to sustain it.

In preparing for the nationwide launch, the BreastCancerTrials.org team made improvements to the software based on lessons from this study. New features included the ability of users to select a health history form customized to their situation (having a new diagnosis vs metastatic vs posttreatment survivor); to use BreastCancerTrials.org as an anonymous guest; to register for a Trial Alert Service; and to browse through BreastCancerTrials.org listings without creating a profile. The trial coding interface and matching engine (caMatch) now broker more complex eligibility criteria. Although trials are still entered by BreastCancerTrials.org staff, the trial coding process is now more efficient. The nationwide service launched in October 2008 [27] and currently lists over 500 trials. Patients who wish to use this or other clinical trial matching websites should keep copies of their medical records so that they can enter a profile that will facilitate their gaining access to appropriate opportunities to participate in research studies. Initiatives aimed at providing patients with treatment summaries, especially if integrated with electronic health records, will make it easier for patients to use online resources such as BreastCancerTrials.org.

## Abbreviations

HER2/neu: human epidermal growth factor receptor 2
USCF: University of California, San Francisco
